# Natural Mutation in Naked Mole‐Rat UCP1 Refutes Importance of the Histidine Pair Motif for Proton Conductance and Thermogenesis

**DOI:** 10.1111/apha.70109

**Published:** 2025-09-24

**Authors:** Michael J. Gaudry, Amanda Bundgaard, Maria Kutschke, Klaudia Ostatek, Margeoux A. S. Dela Rosa, Paul G. Crichton, Jane Reznick, Martin Jastroch

**Affiliations:** ^1^ Department of Molecular Biosciences The Wenner‐Gren Institute, Stockholm University Stockholm Sweden; ^2^ Department of Biology—Zoophysiology Aarhus University Aarhus Denmark; ^3^ Biomedical Research Centre, Norwich Medical School University of East Anglia Norwich UK; ^4^ University of Cologne, Faculty of Medicine and University Hospital Cologne Cluster of Excellence Cellular Stress Responses in Aging‐Associated Diseases (CECAD) Cologne Germany

## Abstract

**Aim:**

Uncoupling protein 1 (UCP1) is the crucial protein for non‐shivering thermogenesis in placental mammals, but the molecular mechanism of thermogenic proton transport is still unknown. Its histidine pair motif (H145 and H147) has been claimed as a critical element for proton translocation, leading to the paradigmatic “cofactor model” of the UCP1 thermogenic mechanism. The histidine pair motif is mutated (H145Q) in the naked mole‐rat (NMR, *Heterocephalus glaber*) UCP1, suggesting disrupted thermogenic function in line with NMR's poor thermoregulatory abilities. Here, we investigated the functionality NMR versus mouse UCP1 to scrutinized the importance of the histidine pair motif.

**Methods:**

Respiratory analyses for UCP1 function were performed in isolated brown adipose tissue mitochondria from NMR and mouse. The histidine pair motif of NMR UCP1 was manipulated through mutations, ectopically overexpressed in HEK293 cells and subjected to plate‐based respirometry for functional comparison.

**Results:**

Isolated BAT mitochondria of NMRs display guanosine diphosphate‐sensitive respiration, indicative of thermogenically competent UCP1. Overexpressed wildtype NMR UCP1 demonstrates proton leak activity comparable to mouse UCP1. Neither restoration of the histidine pair motif nor full ablation of the motif through a double mutation affects UCP1‐dependent respiration.

**Conclusions:**

The UCP1 variant of the NMR, a warm‐adapted fossorial species, excludes the histidine pair motif as crucial for UCP1 thermogenic function. Collectively, we show that functional investigation into natural sequence variation of UCP1 not only casts new light on the thermophysiology of NMRs but also represents a powerful tool to delineate structure‐function relationships underlying the enigmatic thermogenic proton transport of UCP1.

## 1 Introduction

Brown adipose tissue (BAT) is the major site for adaptive nonshivering thermogenesis (NST) in placental mammals, enabling the maintenance of high body temperatures in the cold [1]. This unique tissue is optimized for systemic thermogenesis, displaying dense vascularization for nutrient delivery, gas exchange, and heat transport, as well as substantial sympathetic innervation for central regulation. Brown adipocytes display multilocular lipid droplets that facilitate lipid mobilization and are densely packed with mitochondria of high oxidative capacity.

The thermoeffector protein, uncoupling protein 1 (UCP1), is highly expressed in BAT, and localized to the mitochondrial inner membrane where it is induced by endogenous free fatty acids (FFAs) of various lengths (> 8 carbon) [2], or artificially with retinoic acid analogs (e.g., arotinoid acid [TTNPB]; [3]) and even commonly used drugs such as ibuprofen [4]. UCP1 is inhibited by purine (e.g., guanosine or adenosine di‐ or triphosphates [GDP, GTP, ADP, ATP]) or pyrimidine [5] nucleotides. Once activated, UCP1 catalyzes proton leak across the mitochondrial inner membrane, leading to futile proton cycling and increased rates of substrate oxidation and heat production [6, 7]. The mechanism by which UCP1 facilitates proton transport from the intermembrane space to the matrix remains enigmatic, with at least four competing hypotheses [8], despite the protein structure being recently revealed. The cryo‐EM structures of UCP1 in the nucleotide‐free state, bound to the inhibitors GDP, ATP, or UTP, or bound to the chemical uncoupling agent dinitrophenol (DNP), all show the protein in a single conformation called the c‐state, where the open central cavity of the protein is facing the cytosol [5, 9, 10]. In this conformation, the path of protons is not apparent with an impermeable matrix gate blocking access to the mitochondrial matrix [9]. One may expect that, in a similar fashion to the closely related adenine nucleotide translocator (ANT; [11]), UCP1 may use a carrier‐like mechanism and change conformation to an m‐state, providing a pathway for proton conductance [12], but this has yet to be established. Compelling arguments have been put forward that DNP, suggested to induce proton leak via UCP1, is not a surrogate for the natural activator, fatty acids, binding weakly within the central cavity, and failing to induce a proton pathway nor any notable conformational alterations [13]. Notably, DNP does not activate UCP1‐dependent proton leak in respiratory studies of isolated mitochondria utilizing UCP1 KO controls [14]. With this important question on the thermogenic proton conductance pathway, insights into the functional consequences of amino acid substitutions will be key for elucidating structure–function relationships underlying the UCP1 mechanism.

The role of UCP1 has been modulated throughout the course of evolution, with the orthologue in the stem eutherian ancestor gaining thermogenic function [1] and its repeated secondary inactivations in several eutherian species ([15, 16, 17, 18]). Improved thermogenic capabilities may be expected to have evolved among species that must endure cold conditions, while reduced UCP1 functionality may be expected for those that are exposed to little or no thermal challenges due to the accumulation of unfavorable mutations. In most cases, documented UCP1 inactivations correlate with increases in body size (e.g., horses, whales, elephantids, sirenians, and elephant seals; [16, 17]). However, several tropically distributed eutherian groups (e.g., sloths, armadillos, pangolins; [16]) also display UCP1 inactivations, suggesting that relaxed thermoregulatory requirements dictated by ecological niches may reduce selection for UCP1‐mediated NST.

**FIGURE 1:**
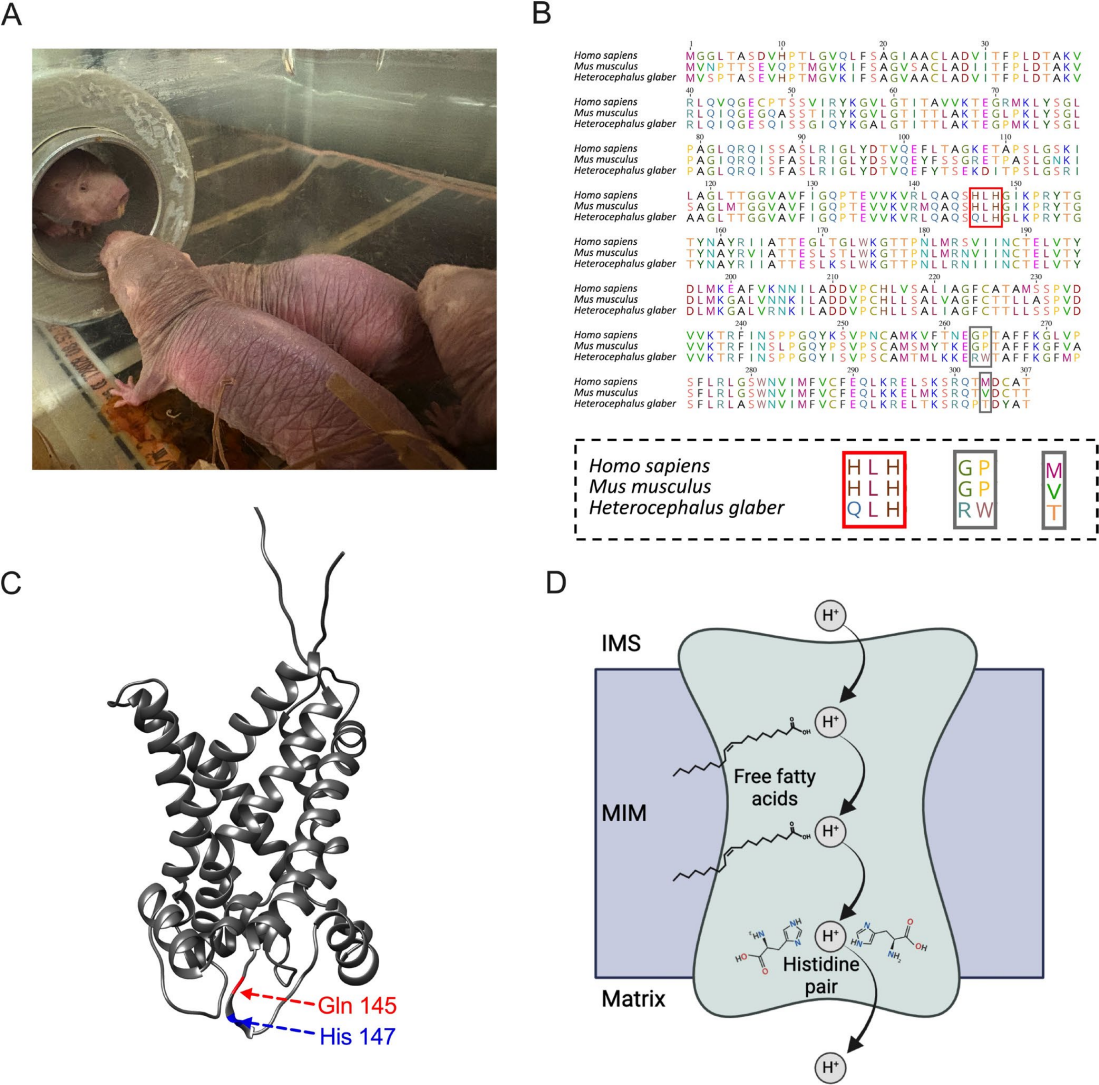
(A) NMRs in laboratory colony—Reznick lab, Cologne Germany. (B) UCP1 amino acid alignment from the naked mole‐rat ( *Heterocephalus glaber* ), mouse ( *Mus musculus* ), and human ( *Homo sapiens* ). The histidine pair motif is indicated with a red box. Gray boxes represent the other unique NMR amino acid substitutions highlighted by Kim et al. [19]. These areas are enlarged below the main alignment. Amino acid sequences were virtually translated from nucleotide sequences with NCBI IDs: AFSB01162372.1; NM_021833.5; AEKQ02007025.1 and aligned with MUSCLE [20]. (C) NMR UCP1 virtually folded with Alphafold 2 and visualized in UCSF Chimera 1.18. Locations of residues at positions 145 and 147 are indicated in red and blue, respectively. (D) Depiction of the “cofactor model” modified from Klingenberg and Huang [21] as a hypothetical mechanism for UCP1 proton transport (Created with BioRender.com). In this proposed mechanism UCP1 binds fatty acids as cofactors to provide protonatable sites via their carboxyl groups allowing protons to “hop” from one protonatable site to the next across the mitochondrial inner membrane (MIM). As histidine amino acids are also protonatable, Klingenberg and Huang [21] proposed that these are the crucial final protonatable sites needed to transfer protons from the intermembrane space (IMS) into the mitochondrial matrix.

The naked mole‐rat (NMR; *Heterocephalus glaber* ; Figure 1A) offers a remarkable model to test the functionality of UCP1 in response to ecophysiological adaptations. NMRs are geographically restricted to equatorial parts of eastern Africa, inhabiting burrow networks that reportedly maintain a highly “thermally buffered” stable ambient temperature year‐round between 30°C and 34°C [22, 23]. NMRs live in eusocial colonies and employ behavioral mechanisms (e.g., huddling) to reduce heat loss during cold exposure [23]. This species displays lower metabolic rates than expected based on its body size [23, 24].

When artificially exposed to various ambient temperatures, NMRs display poikilothermy, allowing their body temperatures to conform to the environment [25]. Scholander plots (metabolic rates against ambient temperature), however, show that NMRs display homeothermy within a very narrow thermoneutral zone of around 31°C–34°C, surrounded by metabolic rate increases above the upper critical temperature to actively dump heat, and below the lower critical temperature, indicative of cold‐induced thermogenesis. Presumably, the lack of fur as insulation (Figure 1A) prevents any maintenance of body temperature, and whether the increased heat dissipation benefits social thermoregulation in the burrow remains to be investigated. Below 28°C–29°C, the NMRs are switching off attempts to counter‐heat and conform with the environment [23, 25], similar to other proto‐endothermic mammals such as tenrecs [26].

Noradrenergic stimulation elicits thermogenic metabolism in the NMR [27], similar to other rodents with BAT. Morphological and anatomical characteristics of BAT are present in the NMR, and ß3 adrenergic inhibition indicates that the increases in NST are localized in BAT [28]. However, the authors suggest that BAT‐mediated thermogenesis in NMRs produces inadequate heat to maintain high body temperatures when NMRs are exposed to 20°C, possibly hinting at a reduced functionality of UCP1 or BAT. Furthermore, BAT thermogenesis in the NMR diminishes under hypoxia, which is possibly favorable to reduce oxygen depletion in fossorial environments [29], although NMRs are well known to be extremely hypoxia tolerant [30].

Given these previous findings, we hypothesized that NMRs may display reduced reliance on UCP1‐mediated thermogenesis under natural conditions, which, throughout the course of evolution, led to reduced selection pressure and possibly the accumulation of unfavorable amino acid substitutions for UCP1 functionality. Upon sequencing the NMR genome, Kim et al. [19] highlighted unique amino acid substitutions among eutherian mammals [19] Figure 1B). The authors suspected that these mutations reflect the distinct thermoregulatory strategy of the NMR and UCP1 function. Of particular note, the H145Q substitution occurs at the first site of the “histidine pair motif”. This well‐known motif at amino acids 145 and 147, localized to a matrix side loop of the protein that links transmembrane alpha helix 3 and the small matrix helix h34 (Figure 1C depicts the location of the histidines in a structural model), is largely conserved as a histidine‐leucine‐histidine (HLH) sequence in eutherian mammals (Figure 1B) and has been previously identified as a key site for thermogenic function [31]. In this prominently cited work of the Klingenberg laboratory, the authors introduced mutations in the UCP1 histidine pair motif of the golden hamster ( *Mesocricetus auratus* ), overexpressed UCP1 variants in yeast mitochondria, and reconstituted the protein in proteoliposomes. Individual H145Q and H147N mutations reduced proton conductance by ~90% compared to wildtype UCP1 [31]. Remarkably, UCP1 activity was essentially abolished with combined H145Q and H147N mutations.

Klingenberg and Huang [21] proposed a mechanistic model that explains the structure–function significance of the “histidine pair motif” whereby UCP1 utilizes FFAs as cofactors to transport protons across the mitochondrial inner membrane, termed the “cofactor model”. Carboxyl groups of the FFAs are protonatable and exposed in the central cavity of the protein. Protons are translocated from one protonatable site to the next along the inner core of UCP1. Histidines, with their imidazole side chains, have the ability to act as proton donor/acceptor sites and, at positions 145 and 147, are proposed by Klingenberg and Huang [21] as the final protonatable site before protons are transferred to the mitochondrial matrix (Figure 1D). Notably, these mutational experiments by Bienengraeber et al. [31] have been broadly accepted, and the resulting model provided the rationale for many further mechanistic studies on UCP1.

In this study, we find that the purine nucleotide GDP inhibits respiration of isolated BAT mitochondria from NMR, as from mouse, indicative of a thermogenically competent UCP1 variant. To delve into the functional characterization of NMR UCP1, we overexpressed the protein in human embryonic kidney cells (HEK293) and performed plate‐based respirometry to assess thermogenic functionality. Mutations were introduced to restore the histidine pair motif (Q145H), then to mimic the single H147N mutation, and fully ablate the motif with the H145Q and H147N double mutant. Our results show that the NMR UCP1 variant is thermogenically competent but, in contrast to previous claims, the histidine pair motif is not required for proton transport.

## 2 Results

### 2.1 Detection and Quantification of NMR UCP1

**FIGURE 2:**
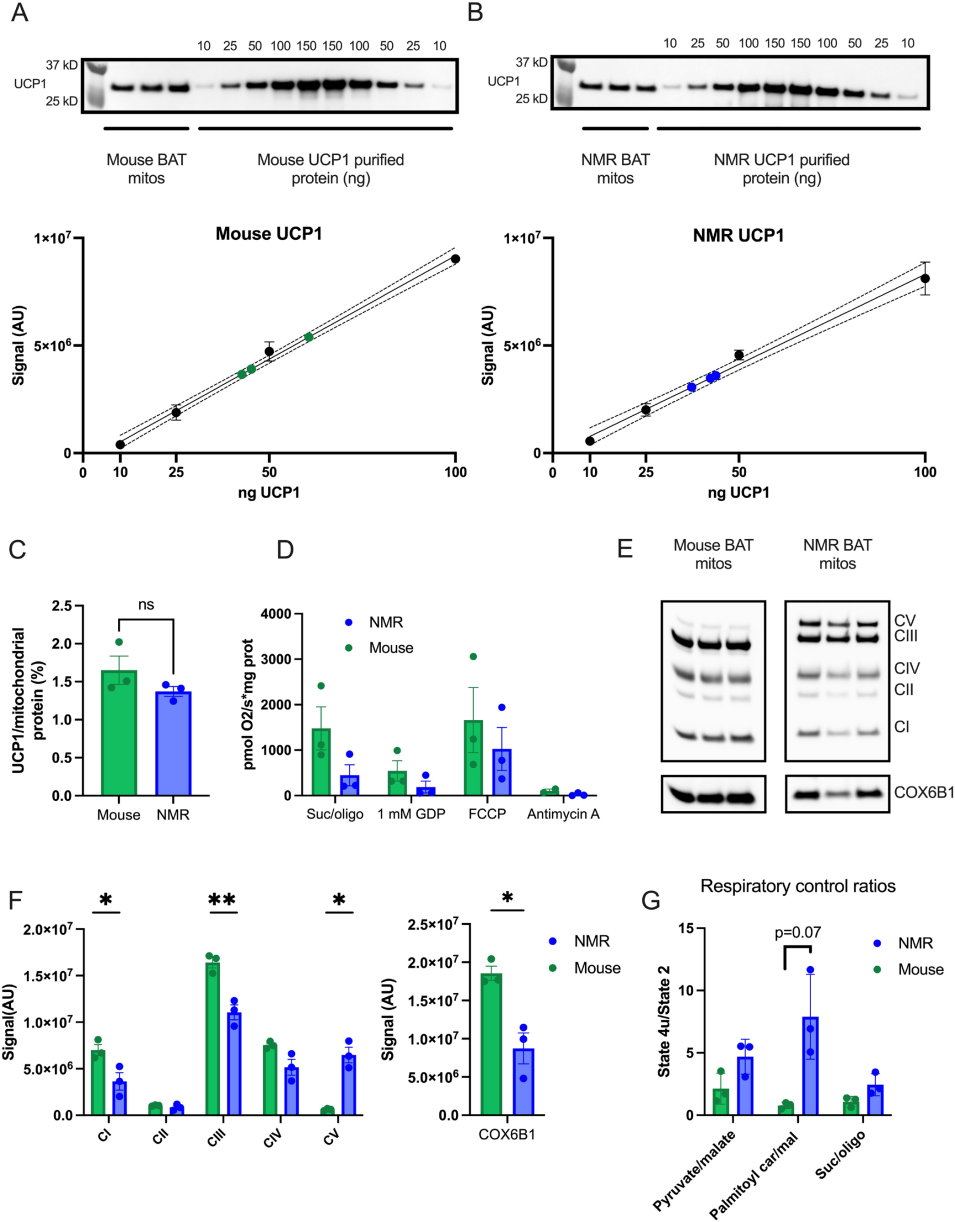
(A, B) Immunoblotting of UCP1 (anti‐UCP1 primary antibody: R&D Systems MAB6158 and anti‐mouse secondary antibody: AP130P (EMD Millipore Corp.)) from isolated mouse and NMR BAT mitochondria samples alongside purified recombinant mouse and NMR UCP1 standards. Ponceau S images can be seen in Figure S2. Standard means are plotted as black dots ±SD, whereas UCP1 levels from mitochondrial samples are shown in green (mouse) or blue (NMR). UCP1 levels were interpolated from a simple linear regression among the standards. The solid line indicates the regression line and the dotted lines represent the 95% confidence interval. Standards ranged from 10 ng to 150 ng of UCP1, but the 150 ng bands were outside of the lineage range, and thus excluded from the linear regression analyses. (C) Calculated UCP1 levels from mouse and NMR brown fat mitochondria as a percentage of total mitochondrial protein. No significant difference was found using a Welch's unpaired *t*‐test. Data are presented as means ±SEM (*n* = 3). (D) Chamber‐based respirometry of isolated BAT mitochondria from mice and NMRs. Mitochondria were energized with succinate (suc), in the presence of rotenone, and oligomycin (oligo) to measure the proton leak component of respiration. The mitochondria were then treated with 1 mM GDP, a UCP1 inhibitor. Maximal respiration rates were achieved with FCCP. Finally, injection of antimycin A allowed correction of nonmitochondrial respiration. No significant difference was found using a Welch's unpaired *t*‐test. Data are presented as means ±SEM (*n* = 3). (E) Immunoblotting of mouse and NMR isolated BAT mitochondria for oxidative phosphorylation complex subunits and cytochrome c oxidase subunit 6B1. (F) Densitometric analyses of oxidative phosphorylation complex subunits and cytochrome c oxidase subunit 6B1. Data are presented as means ±SEM (*n* = 3). Welch's unpaired *t*‐test were used to compare the levels between species and significant differences are indicated with *p < 0.05 or **p < 0.01. (G) Respiratory control ratios calculated as the State 4u respiration/State 2 under various substrates.

As animal housing conditions, temperature sensing, and/or age differ between NMRs and mice (see methods section for full details), we first compared the UCP1 protein concentrations in the isolated mitochondria of NMR and mouse BAT using immunoblotting. The identical avidity of the UCP1‐specific antibody to the epitopes of both NMR and mouse UCP1 cannot be assumed. Therefore, the Western blot analysis was performed alongside purified recombinant mouse and NMR UCP1 mass standards that had been isolated from bacterial inclusion bodies (Figure 2A,B). Linear regression analyses revealed that NMR UCP1 levels ranged between 1.24% and 1.46% of total mitochondrial protein (37.3–43.8 ng in a 3 μg mitochondrial sample), while the mouse UCP1 levels were not significantly different, quantified as 1.42%–2.02% of total mitochondrial protein (42.6–60.6 ng in a 3 μg sample; Figure 2C).

### 2.2 GDP‐Sensitive Respiration in Native BAT Mitochondria of NMR

Next, the BAT mitochondria were subjected to chamber‐based respirometry (Figure 2D). Using succinate as the substrate, the addition of 1 mM GDP potently inhibited respiration in NMR mitochondria by 260 pmol O_2_/s*mg protein (65.8%) and in mouse mitochondria by 934 pmol O_2_/s*mg protein (64.9%; Figure 2D), respectively. FCCP‐induced respiration rates (state 4u) in NMR mitochondria, though not significantly different, suggested lower substrate oxidation capacity compared to mouse mitochondria. Indeed, the immunological detection of respiratory chain complex subunits revealed lower levels for complex I and complex III subunits, as well as COX6B1 in NMR BAT mitochondria, assuming similar antibody avidities (Figure 2E,F). Notably, ATP synthase subunit detection levels were higher in NMR compared to mouse mitochondria (Figure 2E,F).

The differences in substrate oxidation capacity will inevitably affect the magnitude of uncoupled respiration [32], rendering the differences in GDP‐sensitive respiration inappropriate to compare the potency of UCP1 activity between NMR and mouse UCP1. Estimating the coupling state of the mitochondria using the respiratory control ratio (RCR) of state 4u/state 2 succinate respiration reveals a ratio close to 1 in mouse BAT mitochondria, caused by UCP1 activity due to residual fatty acids, while NMR mitochondria under identical measuring conditions displayed RCRs of ~2 (Figure 2G). With residual NMR and mouse mitochondria, we collected respiration data using pyruvate/malate and palmitoyl carnitine (Figure S1), where the RCRs were similarly low in mouse mitochondria, but in NMR mitochondria, ~5 for pyruvate/malate and ~8 for palmitoyl‐carnitine (Figure 2G), altogether suggesting less basal activity of NMR UCP1, possibly due to lower fatty‐acid sensitivity of NMR UCP1‐mediated proton conductance.

### 2.3 NMR UCP1 Function in Overexpressing HEK293 Cells

**FIGURE 3:**
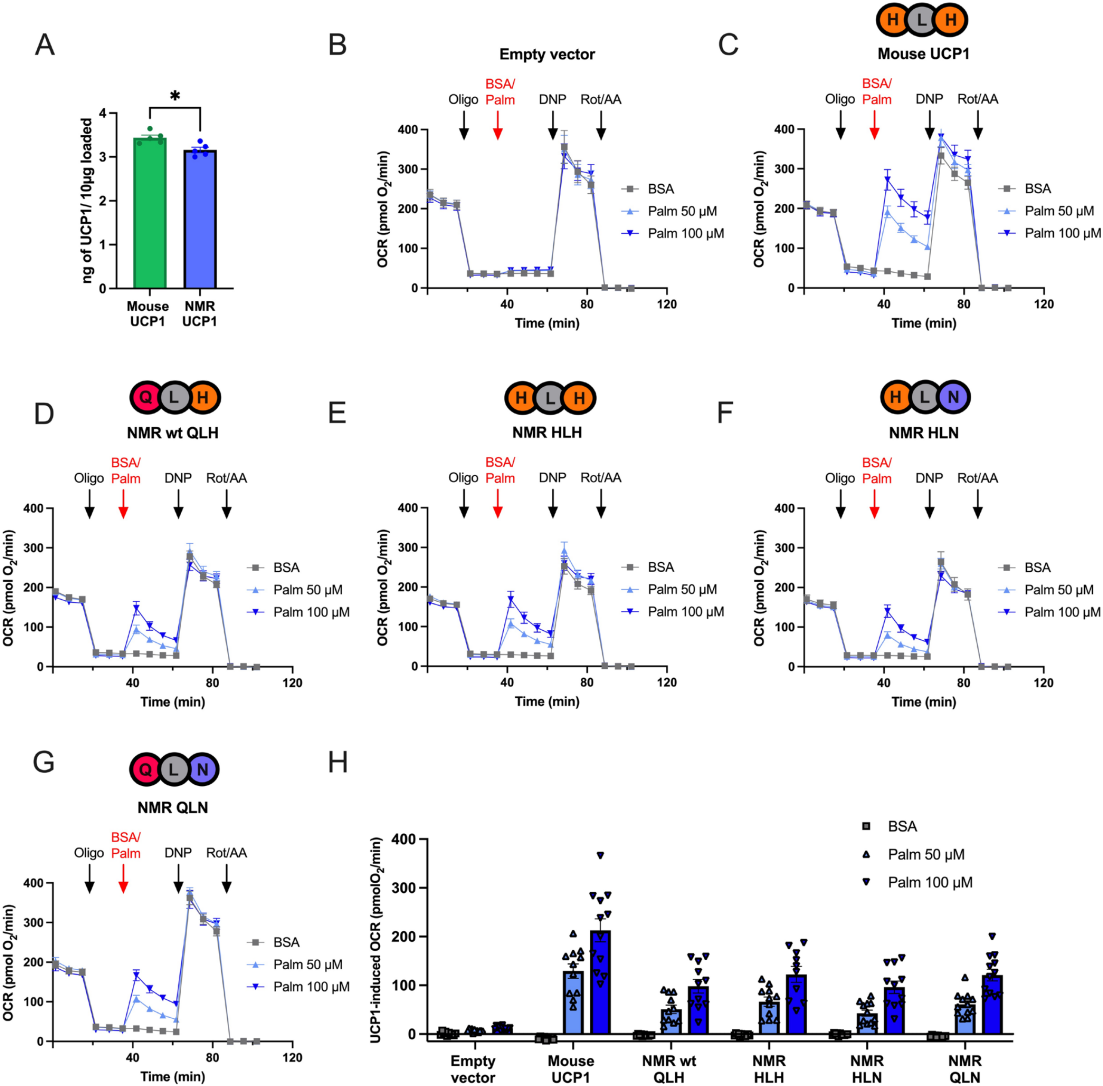
(A) UCP1 levels from stable HEK293 cell lines quantified using a monoclonal UCP1 antibody (R&D Systems MAB6158) and purified protein standards (Figure S3). Data are presented as means ±SEM (*n* = 5). Welch's unpaired *t*‐test were used to compare the levels between species and significant differences are indicated with * (*p* < 0.05). (B–G) Plate‐based respirometry traces of HEK293 cells transiently transfected with an empty pcDNA3.1 vector (B), mouse UCP1 (C), and naked mole‐rat wildtype UCP1 (D). The amino acid identity of the histidine pair motif is indicated above the traces. Points at which oligomycin (oligo), DNP, and rotenone+antimycin A (rot/AA) were injected are indicated. Red arrows denote BSA vehicle control or palmitate (palm; UCP1 activator) injections (end concentration: 50 or 100 μM). Traces have been corrected for nonmitochondrial respiration. NMR histidine pair mutants were tested as well, where the histidine pair was restored as “HLH” (E), the H147N mutation was introduced (“HLN”; F), or the histidine pair was ablated (“QLN”; G). (H) OCR responses to BSA (vehicle control) or palmitate calculated as the average of the first two measurement points after injection minus the three measurement points after the oligomycin injection. Plate‐based respirometry data are presented as means ±SEM (*n* = 9–12 from 3 independent runs).

Next, we generated HEK293 cell lines stably overexpressing NMR UCP1, mouse UCP1 (positive control), and empty pcDNA3.1 vector (negative control). HEK293 cells overexpressing mouse UCP1 displayed slightly higher UCP1 levels than NMR UCP1‐expressing cells (Figure 3A; Figure S3), but both were calculated to be ~0.03% of total protein. Plate‐based respirometry of intact HEK293 cells revealed dose‐dependent increases in oxygen consumption rates (OCRs) upon adding palmitate, an established UCP1 activator, to NMR UCP1 and mouse UCP1‐expressing cells, when compared to control cells (Figure 3B–D), suggesting that native NMR UCP1 is activatable with free fatty acids. Mouse UCP1‐expressing HEK cells displayed higher activatability than NMR UCP1 with both palmitate (Figure 3C,D) and the retinoic acid analog, TTNPB (Figure S4), but differences in maximal respiration rates may affect potential species differences between mouse and NMR in line with the results from BAT mitochondria. Nevertheless, the NMR wildtype UCP1 variant demonstrates that H145 of the histidine pair motif is not required for proton translocation by UCP1.

Next, we mutated the NMR UCP1 sequence by introducing the missing histidine (Q145H, labeled HLH), anticipating higher proton leak respiration, and ablating only histidine 147 (H147N, labeled HLN), as well as ablating both histidines (Q145, N147, labeled QLN), anticipating loss of function. Despite our expectations, neither gain nor loss of the histidine pair motif changed inducible respiration of NMR UCP1 (Figure 3D–H), demonstrating (a) that the histidine pair is unnecessary for UCP1 protonophoric function, which is in stark contrast to previous claims [31], and (b) that the loss of the histidine pair does not contribute to lower inducibility of the NMR UCP1 compared to the mouse variant. All plate‐based respirometry experiments with stably transfected HEK293 cells were also performed using TTNPB as a UCP1 activator instead of palmitate but provided similar results (Figure S4).

### 2.4 Inhibition and Activation of UCP1 in Permeabilized Cells

**FIGURE 4:**
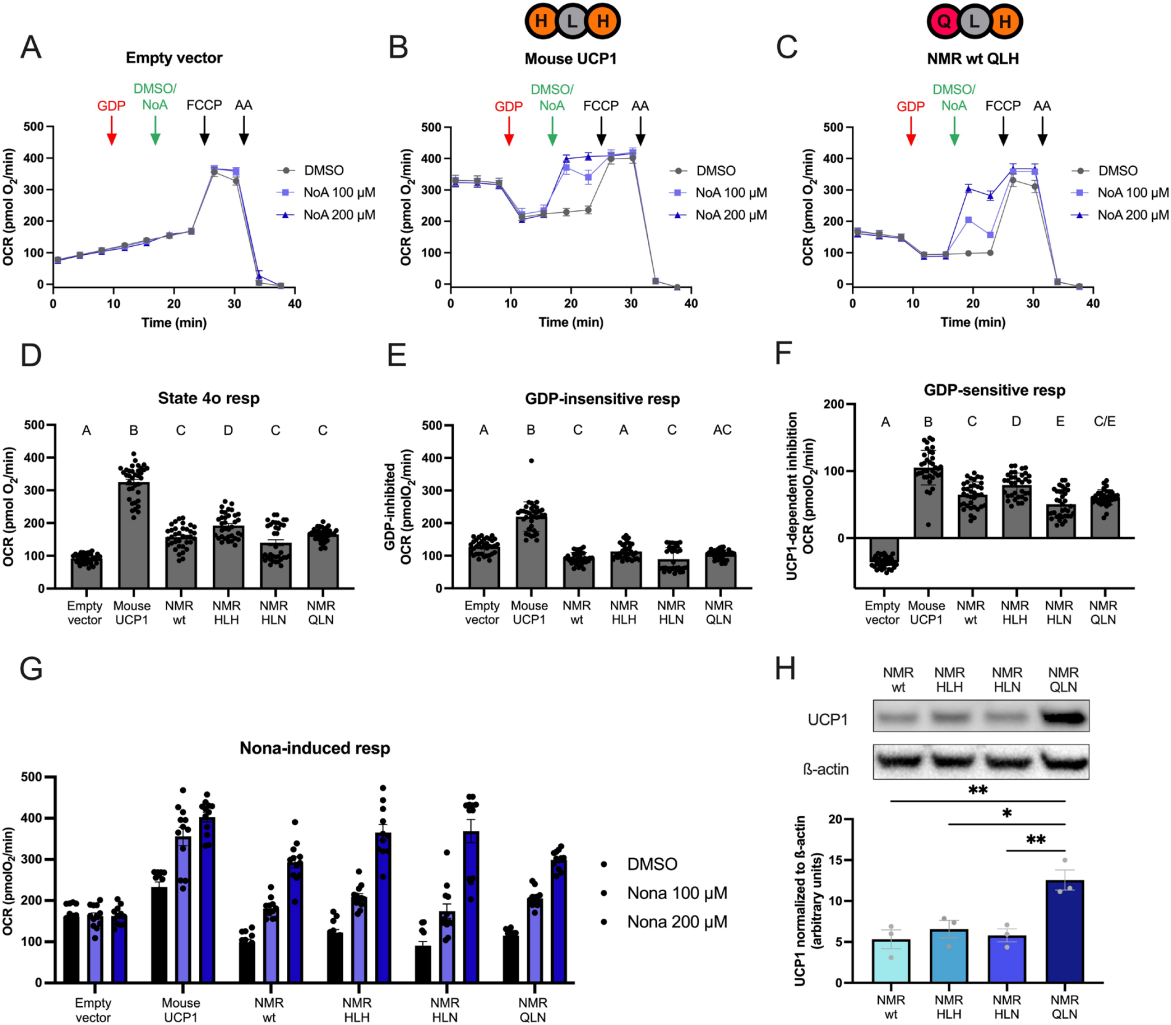
Plate‐based respirometry of permeabilized HEK293 cells stably expressing an empty pcDNA3.1 vector (A), mouse UCP1 (B) or naked mole rat wildtype UCP1 (C). The amino acid identity of the histidine pair motif is indicated above the traces. In these traces, state 4o respiration in the presence of succinate is measured, followed by an injection of GDP to inhibit UCP1 (red arrow). Injection of DMSO (vehicle control) or nonanoic acid (NoA; end concentration: 100 or 200 μm) is used to activate UCP1 (green arrow). The maximal respiration is achieved with injection of FCCP, then injection of antimycin A (AA) allows for the correction for nonmitochondrial respiration. Traces from all histidine pair mutants are shown in Figure S5. OCRs of state 4o respiration (D), and both GDP‐insensitive (E) and GDP‐sensitive (F) respiration have been compared using ordinary one‐way ANOVAs. Different statistical groups (*p* < 0.05) are indicated with different letters. Responses to DMSO or nonanoic acid are shown in (G). Data are presented as means ±SEM (*n* = 10–12 from 3 independent runs). Representative western blot using polyclonal UCP1 antibody (Abcam ab155117) across stably expressing NMR histidine pair UCP1 variants (H). Densitometric analyses are below and have been analyzed with an ordinary one‐way ANOVA and statistically significant differences indicated with *p < 0.05 or **p < 0.01. Data are presented as means ±SEM (*n* = 3).

To investigate the inhibition and activation of UCP1 directly, we permeabilized stably expressing HEK293 cells. Using succinate as substrate in the presence of 0.4% defatted bovine serum albumin (BSA), NMR UCP1 mitochondrial displayed higher respiratory control (state 4u/state 4o respiration rates) than mouse UCP1‐containing mitochondria (Figure 4A–F), further corroborating the lower uncoupling activity of NMR UCP1 under identical conditions. Between the NMR mutants, respiration rates were similar. The administration of 500 μM GDP potently inhibited all UCP1 variants, seen as a similar reduction of UCP1‐dependent, GDP‐sensitive respiration (Figure 4A–F). The UCP1 variants were then activated with a medium‐chain fatty acid, nonanoic acid, showing dose‐dependent increases in OCRs. With 100 μM nonanoic acid, cells expressing mouse UCP1 displayed the greatest response, and all NMR variants displayed similar responses to the NMR wt protein (Figure 4G). Taken together, the permeabilized cells support the lower inducibility of NMR UCP1 compared to mouse UCP1 and that the histidine motif is obsolete.

The expression levels of stably UCP1 expressing clones can vary. While we noted similar UCP1 levels in the stably expressing HEK293 cell lines among wildtype NMR (wt QLH), NMR HLH, and NMR HLN variants, the stable QLN mutant contained twice as much UCP1 (Figure 4H), which could be detected using a polyclonal UCP1 antibody.

### 2.5 Transient Overexpression of UCP1

**FIGURE 5:**
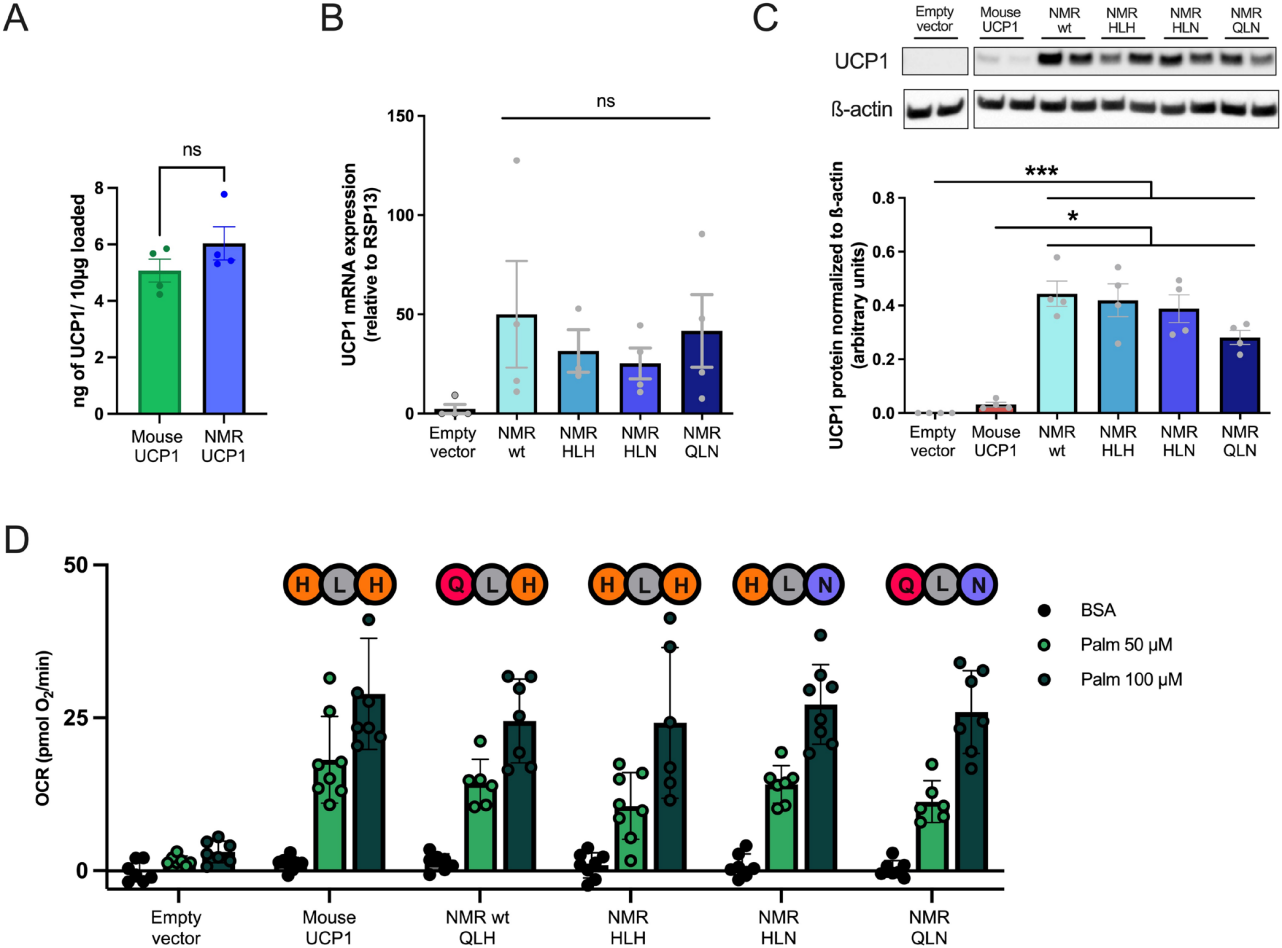
(A) Calculated UCP1 protein levels between HEK293 cells transiently transfected with mouse and NMR wildtype UCP1 as determined by immunoblotting with a monoclonal UCP1 antibody (R&D Systems [MAB6158]) and purified UCP1 standards (Figure S6; *n* = 4). (B) mRNA levels of naked mole‐rat UCP1 among variants relative to RSP13. mRNA from cells transfected with the empty vector (Emp) were included in the experiment as a negative control. (C) Representative Western blot using polyclonal UCP1 antibody (Abcam [ab155117]), which could detect all NMR variants. Densitometric analyses of UCP1 relative to ß‐Actin are below. No significant differences were found between HEK293 cells transfected with NMR variants (*n* = 4) when using ordinary one‐way ANOVA with Tukey's post hoc test. (D) Palmitate‐induced respiration (50 and 100 μM; or BSA control solution) among HEK293 cells transiently transfected with the empty vector, mouse UCP1, NMR wt UCP1, as well as the NMR histidine pair mutants (*n* = 7–9 per group and from 4 independent runs). Full plate‐based respirometry traces of transiently transfected HEK293 cells are shown in Figure S7. Data are presented as means ±SEM.

Although the activator responses of UCP1 overexpression are generally lower when transient transfection is used, the identical transfection treatment greatly reduces variation in UCP1 content ([17]). The transient transfection of NMR UCP1 mutants resulted in similar mRNA and protein levels across all variants, including the histidine double mutant QLN (Figure 5A–C and see Figure S7 for full plate‐based respirometry traces). If at all, the QLN variant protein levels are slightly lower (Figure 5C), but the activator responses are similar to all other UCP1 variants (Figure 5D and Figure S7).

## 3 Discussion

Here, we demonstrate that despite the seemingly poor thermoregulatory abilities of the NMR [25], UCP1 of this species is thermogenically active. The fatty‐acid sensitivity of proton conductance appears to be slightly lower than that of mouse UCP1, in line with the weaker thermoregulatory abilities of this species. We delineate with this naturally occurring variant that the prominent UCP1 histidine pair motif, previously considered essential for thermogenic proton transport, is obsolete. Moreover, the repair of the motif towards the full histidine pair does not improve fatty acid‐induced proton transport, and this unique mutation in the NMR UCP1 is therefore not responsible for lower activity rates.

Previously, experiments in the NMR noted NST upon noradrenaline injection [27], and the administration of the ß3‐adrenergic receptor antagonist SR59230A increased cooling rates upon cold exposure, both suggestive of thermogenic BAT in this species [28]. Others have reported on blunted BAT thermogenesis in hypoxia and immunological detection of UCP1 in BAT [29]. Adding further evidence for BAT thermogenesis in NMR, our study shows GDP‐sensitive respiration in isolated BAT mitochondria, indicative of a functionally thermogenic UCP1 variant. When comparing UCP1 function between mouse and NMR, it is of paramount importance to control for protein content in the two different species. Immunological detection per se is not directly attributable to UCP1 levels, as the epitope recognition between species may differ. However, after carefully evaluating the immunodetection of the UCP1 variants using purified recombinant standards to normalize signals with a UCP1‐specific monoclonal antibody, we demonstrate that isolated BAT mitochondria of our experimental NMRs and mice displayed similar UCP1 levels, enabling us to directly compare UCP1 functionality. The comparative functional data suggest that NMR UCP1 has slightly lower activator (e.g., fatty acids) sensitivity than its mouse counterpart, based on higher respiratory control of NMR mitochondria irrespective of substrate, which is in line with less developed thermogenic capacities in NMRs. The overexpression of comparable amounts of NMR and mouse UCP1 in HEK293 cells, providing an identical genetic and cellular background, further corroborated lower activator sensitivity of the NMR UCP1.

In the HEK cell system, we directly tested the importance of the histidine pair motif, which is partially disrupted in the NMR UCP1. Considering the earlier data by Bienengraeber et al. [31] claiming the importance of the histidine pair motif, it was not unreasonable for Kim et al. [19] to surmise that unique mutations to the NMR UCP1 may hinder protein function and reflect the unusual thermoregulation of the species. Yet, our data refute the functional importance of this structural element, as we were unable to recapitulate the results of Bienengraeber et al. [31] that claimed ~90–100% reductions in proton transport upon mutation of the critical histidine residues at positions 145 and 147. Notably, reversion of the natural H145Q mutation to restore the histidine pair motif failed to improve UCP1 activatability, also demonstrating that the histidine pair motif is not responsible for the lower activity of the NMR UCP1. Similarly, the introduction of the H147N mutation also failed to elicit notable differences in UCP1 functionality. Even the UCP1 variant displaying a complete ablation of the histidine pair motif (“QLN” at sites 145–147) maintained sensitivity of activation and GDP inhibition, no matter if long (palmitate) or medium (nonanoic acid) chain fatty acids, or a retinoic acid analog, TTNPB, was used for activation. The natural UCP1 variant in the NMR and these mutational experiments refute the importance of the histidine pair motif, which was proposed as protonatable sites for the “cofactor” mechanistic model of proton leak by UCP1. However, the results of Bienengraeber et al. [31] cannot completely be discounted, as their functional experiments were performed with wildtype and mutant golden hamster ( *Mesocricetus auratus* ) UCP1. Future studies are required to scrutinize the possibility of species‐specific importance of the histidine pair motif.

Future studies to further examine BAT among different NMR colony members (e.g., queens, workers, soldiers) may be interesting. Indeed, Oiwa et al. [28] reported from thermal imaging data that queen NMRs use BAT thermogenesis when isolated at 30°C, which is not seen among subordinates. Previous hypotheses on the role of BAT included a role for reproduction and offspring incubation in proto‐endothermic tenrecs [7], which may be worth investigating in NMRs. Given the relatively stable high temperatures in the burrow, it would also be interesting to examine if UCP1‐mediated NST could be used by all colony members of this fossorial species to maintain elevated burrow or chamber temperatures.

Overall, our study on the NMR UCP1 variant highlights the importance of investigating natural mutations to examine thermoregulatory behavior and overall protein function.

## 4 Methods

### 4.1 Animals

Adult male C57BL/6Nj mice (*Mus musculus*) were kept at 20°C–24°C in 12 h light/dark cycles and fed *ad libitum* chow diet. Naked mole‐rats ( *Heterocephalus glaber* ) were kept in individual colonies in a system of cages connected with plastic tubes within a humidified incubator (50%–60% humidity, 28°C–30°C), in normal room air and darkness except during feeding and cleaning. Their diet consisted of fresh vegetables, fruit, and tubers (sweet potatoes) *ad libitum*. All water requirements were obtained from the food sources. Male and female nonbreeding (subordinate) naked mole‐rats were used in experiments since subordinate animals do not undergo sexual development or express sexual hormones. The ages selected for this study allowed for physiological age matching such that all animals were at equivalent percentages of maximum lifespan and therefore not the same chronological age (~10‐week‐old mice vs. 7‐year‐old NMRs). All animal procedures were conducted in accordance with European, national, and institutional guidelines and protocols were approved by local government authorities Landesamt für Natur, Umwelt und Verbraucherschutz Nordrhein‐Westfalen (LANUV). License number for mice: UniKoeln_Anzeige§4.20.025. License number for NMRs: UniKoeln_Anzeige§4.21.014.

### 4.2 Isolation of BAT Mitochondria From NMR and Mice

Prior to BAT mitochondrial isolation, both the mice and NMRs were removed from their enclosures during the morning light phases to be euthanized at room temperature after having *ad libitum* access to food. Mitochondria were isolated from BAT interscapular depots as previously described by Mzilikazi et al. [33]. Briefly, the tissues were dissected and placed directly on ice in isolation buffer (250 mM sucrose, 10 mM TES, 2 mM EDTA, 0.5% BSA, pH 7.2). The tissues were finely minced with scissors and razor blade in a petri dish on ice before being disrupted in a glass homogenizer with a Teflon pestle. The homogenate was filtered through nylon mesh and centrifuged at 8740 × *g* for 10 min at 4°C. Supernatant was removed along with lipids from the centrifuge tubes. The pellets were resuspended in isolation buffer B (250 mM sucrose, 10 mM TES, 1 mM EDTA, 0.5% BSA, pH 7.2) using a pipette with a cut‐off tip to avoid too much shearing force before centrifuging at 950 × *g* for 10 min at 4°C. The supernatant was decanted into a fresh tube and centrifuged at 8740 × *g* for 10 min at 4°C. The pellet was then resuspended in isolation buffer C (100 mM KCl, 20 mM TES, 1 mM EGTA, pH 7.2), centrifuged at 8740 × *g* for 10 min at 4°C, and resuspended in a small volume of isolation buffer C. Mitochondrial protein concentrations were established using BCA assay.

### 4.3 Chamber‐Based Respirometry

Mitochondrial oxygen consumption rates were measured with an Oxygraph‐2 k system (Oroboros Instruments, Innsbruck, Austria) at 32°C, which represents the maximum body temperature of naked mole‐rats. Either 0.5 mL or 2 mL chambers were used to measure 5–50 μL of mitochondria. The assay buffer consisted of 50 mM KCl, 5 mM TES, 2 mM MgCl_2_ 6xH_2_O, 4 mM KH_2_PO_4_, 1 mM EGTA, 0.5% BSA, pH 7.2 adjusted with KOH. The yield of mitochondria was sufficient to measure respiration rates in the presence of three substrates: (1) 2 mM malate +5 mM pyruvate, (2) 10 μM palmitoyl carnitine, (3) 20 mM succinate +0.5 μM rotenone +1 μM oligomycin. With the first two substrates, state 2 respiration rates were measured for 1–3 min, followed by injection of 1 mM ADP to induce state 3 respiration. Oligomycin (1 μM) was then added to inhibit ATP synthase, followed by successive injections of FCCP until maximal respiration rates were achieved. Antimycin A was then injected to correct for nonmitochondrial respiration. When performing measurements in the presence of succinate/rotenone/oligomycin, basal respiration rates were followed by an injection of GDP (1 mM) to inhibit UCP1‐dependent respiration, followed by successive injections of FCCP, and finally antimycin A. The mitochondrial oxygen consumption rates were then normalized to mg mitochondrial protein.

### 4.4 UCP1 + pcDNA3.1 Constructs

UCP1 coding sequences designed from the AFSB01162372.1 genomic sequence were synthesized (Basegene) and cloned into pcDNA3.1 vectors using Xhol and Xbal restriction sites. pcDNA3.1 + UCP1 vectors were transformed in DH5 alpha *E. coli* as per the manufacturer's protocol and plated at various concentrations on LB agar + ampicillin (concentration) 10 cm dishes and grown overnight at 37°C. The next day, liquid cultures were prepared in LB broth + ampicillin (100 μg/mL) were inoculated with selected colonies and grown overnight at 37°C while shaking at 225 rpm. Plasmids were then purified from bacterial cultures using a QIAprep spin Miniprep kit (Qiagen) according to the manufacturer's directions, eluted in nuclease‐free water, and sequenced to ensure the proper identity of the UCP1 inserts. Quantification of the purified plasmids was performed using a NanoDrop One Microvolume UV–Vis spectrophotometer (Thermo Scientific).

### 4.5 HEK293 Cell Culture

Human embryonic kidney (HEK293) cells were cultured on T75 or T175 flasks in growth medium consisting of DMEM (high glucose; Gibco) supplemented with 10% fetal calf serum (Gibco) and 1% penicillin/streptomycin ([100 U/mL] Thermofisher). Cells were grown in an incubator at 37°C and 5% CO_2_ and trypsinized at 85%–90% confluency using Trypsin‐EDTA (0.05%) with phenol red (Gibco).

### 4.6 Transient Reverse Transfection of HEK293 Cells

HEK293 cells were trypsinized and counted in duplicate using 0.4% Trypan blue (Bio‐Rad) and a TC20 automated cell counter (Bio‐Rad). The cells (1.76 × 10^6^) with the volume adjusted to 3.2 mL of growth medium were then transferred to 15 mL Falcon tubes. Transfection mixtures were prepared in 1.5 mL tubes consisting of 900 μL DMEM, 9 μg of plasmid DNA, and 36 μL of Polyfect transfection reagent (Qiagen). Transfection mixtures were incubated for 15 min at room temperature before being added to the Falcon tubes containing the HEK293 cells and mixed. The cells were then seeded on Seahorse XF96 microculture plates that had been coated in polyethyleneimine (PEI) at a density of 30 000 cells per well. Leftover cells were seeded on 12‐well plates for RNA and protein isolation.

### 4.7 Stable HEK293 Cell Transfections and Selections

HEK293 cells were seeded on a 6‐well plate at a density of 100 000 per well. The next day, when the cells reached ~60% confluency, the transfection mixture was added to the cells containing 200 μL of DMEM, 2 μg plasmid DNA, and 8 μL Polyfect transfection reagent (Qiagen). The cells were incubated at 37°C and 5% CO_2_ for 24 h, at which point they were washed and fresh growth medium containing penicillin/streptomycin was added. After 2 days, the cells were trypsinized and seeded on 15 cm dishes, the medium was changed, and this time replaced with medium containing Geneticin (G418 [500 μg/mL]; Gibco). The enrichment process lasted for 10 days (cells were split and reseeded 2–3 times during this period). The cell lines were then trypsinized and seeded at a density of 2000 cells per 15 cm dish with maintenance medium containing 10% fetal calf serum and 50 μg/mL Geneticin. After growing the cells for 7 days (changing the medium every 2–3 days), single colonies were selected using cloning disks and transferred to 48 well plates. Colonies were then expanded over several passages and screened for UCP1 expression using qPCR and Western blotting.

### 4.8 Intact Cell Plate‐Based Respirometry of Stable and Transiently Transfected Cells

On the day of the experiment, 24 h after seeding, the cells were washed twice with XF assay base medium (Agilent) supplemented with 10 mM glucose, 10 mM pyruvate, and 2 mM glutamine, and 0.4% bovine serum albumin (BSA), and the pH adjusted to 7.4. The cells were incubated at 37°C without CO_2_ for 1 h before starting the experiment. Following equilibration of the Agilent Seahorse XF96 sensor cartridge, the experiments were initiated at 37°C using 1 min mix, 2 min wait, and 3 min measure cycles. The basal respiration of the cells was measured over 3 cycles. Oligomycin (4 μg/mL end concentration) was then injected from port A of the sensor cartridge to inhibit ATP synthase and measured over 3 assay cycles. The residual oxygen consumption at this point can be attributed to proton leak and nonmitochondrial respiration. Next, the UCP1 activators were injected from port B, and oxygen consumption rates were measured over 4 assay cycles. A retinoic acid analog and known UCP1 activator [3], TTNPB (4‐[(E)‐2‐(5,6,7,8‐tetrahydro‐5,5,8,8‐tetramethyl‐2‐naphthalenyl)‐1‐propenyl]benzoic acid), was used at concentrations of 15 and 30 μM. Wells were also treated with the vehicle control (dimethylsulphoxide [DMSO]). The free fatty acid, palmitate, was also used to activate UCP1 at concentrations of 50 and 100 μM. The palmitate (1 mM) had been equilibrated with 0.17 mM BSA using the Seahorse Biosciences conjugation protocol. Thus, wells were also treated with the 0.17 mM (stock concentration) BSA vehicle control. Next, 2,4‐dinitrophenol (DNP; 100 μM end concentration) was injected from port C to achieve maximal mitochondrial respiration and measured over 3 assay cycles. Finally, nonmitochondrial respiration was measured over 3 cycles following the inhibition of complexes I and III of the electron transport chain by injecting rotenone (4 μM end concentration) and antimycin A (2 μM end concentration) from port D.

### 4.9 Permeabilized Plate‐Based Respirometry of Stably UCP1 Overexpressing Cell Lines

Permeabilized plate‐based respirometry runs were performed with MAS buffer (220 mM mannitol, 70 mM sucrose, 10 mM KH_2_PO_4_, 5 mM MgCl_2_, 2 mM HEPES, 1 mM EGTA, 0.4% fatty acid free BSA, pH 7.1). HEK293 cell lines stably overexpressing UCP1 had been seeded at a density of 20 000 cells per well on PEI‐coated Seahorse XF96 microculture plates. Growth medium was removed from the cell plates, and the cells were washed twice with MAS buffer. MAS buffer containing 10 mM succinate, 2 μM rotenone, 4 μg/mL oligomycin, and 0.003% digitonin was added to a volume of 180 μL. The cells were then left to incubate for 15 min in a CO_2_‐free incubator at 37°C. The extracellular flux assays were performed at 37°C with one cycle equaling a 30 s mix period, followed by a 30 s waiting period and a 2 min measurement period. Basal (state 4o) respiration was measured for 3 cycles, followed by an injection of GDP (500 μM [well concentration]) were added to inhibit UCP1 activity. OCRs were measured for 2 cycles. Then nonanoic acid (100 μM or 200 μM) or DMSO vehicle control was added to activate UCP1, followed by 2 measurement cycles. FCCP (10 μM) was injected from the third port to achieve maximal respiration, followed by 2 measurement cycles. Antimycin A (2 μM) was added to correct for nonmitochondrial respiration and measured over 2 cycles.

### 4.10 RNA Isolation and qPCR

RNA was isolated from transfected cells seeded on 12‐well plates using QIAzol lysis reagent (Qiagen) according to the manufacturer's instructions. Isolated RNA was quantified using a NanoDrop One Microvolume UV–Vis spectrophotometer (Thermo Scientific). cDNA was reverse transcribed using a Quantitect reverse transcription kit (Qiagen). To determine the UCP1 mRNA levels via qPCR, standard curves were first set up with cDNA concentrations of 20, 10, 5, 1, 0.1, and 0.01 ng/μL to test primer efficiencies. Primer pairs were determined to be within the range of 90–110% efficient. Then qPCR reactions were mixed using 1 μL of cDNA (1:10 diluted from reverse transcription), forward and reverse primers (final concentration 0.5 μM), 2.5 μL SYBR green JumpStart Taq ReadyMix (Sigma‐Aldrich), and filled to a total reaction volume of 5 μL using nuclease‐free water.

Primers used:

Naked mole‐rat UCP1 forward: 5′‐TCGGAGAAAGATATAACACCTAGT‐3′.

Naked mole‐rat UCP1 reverse: 5′‐CAGCTGCGATCCTACTTCC‐3′.

Human RSP13 forward: 5′‐CTTGTGCAACACCATGTGAA‐3′.

Human RSP13 reverse: 5′‐CCCCACTTGGTTGAAGTTGA‐3′.

### 4.11 Protein Isolation and Quantification

Protein was isolated from transfected cells seeded on 12‐well plates by first adding RIPA buffer (150 mM NaCl, 50 mM Tris base, 0.5% sodium deoxycholate, 1% IGEPAL CA‐630, 0.1% sodium dodecyl sulfate, pH 8.0) with HALT protease and phosphatase inhibitor (ThermoFisher Scientific). Plates were kept on ice and scraped with a pipet tip. The lysates were then transferred to 1.5 mL tubes and incubated on ice for 30 min with vortexing every 5 min. Lysates were then centrifuged at 18 000 × *g* in a cooling centrifuge set to 4°C. The supernatant was transferred to fresh tubes while the cell debris pellet was left behind. Subsamples were diluted 4× with nuclease‐free water and quantified using Bradford reagent (Sigma‐Aldrich) on a plate reader measuring absorbance at 595 nm. Protein concentrations were calculated according to a BSA standard curve.

### 4.12 UCP1 Standards

Recombinant mouse and NMR UCP1 standards were prepared in *E. coli* as described by Keipert et al. [1]. Briefly, UCP1 coding sequences of NMR and mouse were cloned into a pMW172 expression vector downstream of a N‐terminal six‐histidine tag. UCP1 isoforms were expressed in the C41 (DE3) *E. coli* strain as inclusion bodies and were isolated as described by Echtay et al. [34]. His‐tagged UCP1 variants were purified under denaturing conditions through affinity chromatography using Ni‐NTA Superflow cartridges (Qiagen) as defined in Keipert et al. [1]. His‐tagged UCP1 isoforms were eluted from cartridges under acidic conditions (100 mM Tris HCl (pH 4.5), 8 M urea, 100 mM NaH2PO4). To remove excess urea, eluates were concentrated to 10% of the initial volume using Vivaspin centrifugal concentrators (MWCO 30000 Da) and resuspended in a 10× volume of buffer 1 (20 mM Tris HCl (pH 8.0), 2% (w/v) sarkosyl). Resulting eluates were concentrated once more to 10% of the initial volume and resuspended in a 10× volume of buffer 2 (20 mM Tris HCl (pH 8.0), 0.5% (w/v) sarkosyl). The samples were then applied to PD10 columns (preequilibrated with buffer 2) to eliminate the remaining urea. Final protein concentrations of UCP1 standards were determined by BCA assay, and the samples were snap frozen prior to storage at −80°C until further use.

### 4.13 Western Blot

Isolated mitochondria (3 μg), protein samples from cell lysates (10 μg), or purified UCP1 standards were mixed with 4× loading dye and 10× reducing agent (Invitrogen). Nuclease‐free water was added to a total volume of 25 μL. Mixtures were then loaded into precast 4%–12% Bolt Bis‐Tris gels (Invitrogen). Precision Plus Protein Dual Color Standards (Bio‐Rad) marker was used. Gels were run according to the manufacturers' recommendations using the Bolt system by Invitrogen. Transfers to nitrocellulose membranes were then performed using the iBlot 2 system (Invitrogen) at 20 V for 7 min. Membranes were then washed in deionized water three times before staining with Ponceau S staining solution. Membranes were then blocked with 5% skimmed milk (Sigma‐Aldrich) solution mixed in Tris‐buffered saline, 0.1% Tween 20 (TBS‐T) buffer. After washing the membranes three times with TBS‐T, they were incubated overnight at 4°C on a rocking platform with an anti‐UCP1 antibody (R&D Systems [MAB6158] or Abcam [ab155117]) that had been diluted 1:1000 in 5% BSA TBS‐T solution. Some membranes were also probed for oxidative phosphorylation complex subunits using (Thermofisher [45–8099]) and cytochrome c oxidase subunit 6B1 (Abcam [ab137089]). The membranes were then washed three times with TBS‐T buffer and a secondary anti‐mouse secondary antibody (EMD Millipore Corp [AP130P]) or anti‐rabbit antibody (Abcam [ab6721]) was applied for 1 h after being diluted 1:20000 in 5% milk + TBS‐T solution. The membranes were then washed 3 times with TBS‐T and imaged in a ChemiDoc MP Imaging System (Bio‐Rad). Some membranes were subsequently stripped using stripping buffer (composed of 15 g glycine, 2 g sodium dodecyl sulfate, and 10 mL Tween 20, pH 2 with deionized water added to 1000 mL) on a rocking platform for 20 min at room temperature. The membranes were then blocked in 5% milk solution for 1 h and a ß‐actin horseradish peroxidase‐linked antibody (sc47778; Santa Cruz) was applied after being diluted 1:20000 in 5% milk + TBS‐T. The membranes were then washed three times in TBS‐T and imaged. Protein quantification analyses were performed using Image Lab 6.1 (Bio‐Rad Laboratories).

### 4.14 Statistics

Statistical tests used have been described in the figure legends and were performed in GraphPad Prism 10.

## Author Contributions

M.J.G. conceived of the project, performed experiments, analyzed the data, and wrote the manuscript. A.B., M.K., K.O., and M.A.S.D.R. performed experiments and analyzed the data. P.G.C. analyzed data and helped perform experiments. J.R. provided the animals. M.J. conceived of the project and wrote the manuscript.

## Conflicts of Interest

The authors declare no conflicts of interest.

## Supporting information


**Figures S1–S7:** apha70109‐sup‐0001‐FiguresS1‐S7.pdf.

## Data Availability

The data that support the findings of this study are available from the corresponding author upon reasonable request.
