# Supplementary material for: Natural Mutation in Naked Mole‐Rat UCP1 Refutes Importance of the Histidine Pair Motif for Proton Conductance and Thermogenesis
Source: Acta Physiol (Oxf). 2025 Sep 24;241(10):e70109. doi: 10.1111/apha.70109 (PMC12458454; doi:10.1111/apha.70109)
Supplement: Supplementary file 1 — Figures S1–S7: apha70109‐sup‐0001‐FiguresS1‐S7.pdf. [file APHA-241-e70109-s001.pdf]

Supplementary Materials for

Natural mutation in naked mole-rat UCP1 refutes importance of the histidine pair motif for proton conductance and thermogenesis

Michael J. Gaudry[1], Amanda Bundgaard[2], Maria Kutschke[1], Klaudia Ostatek[1], Margeoux A.S. Dela Rosa[3], Paul G. Crichton[3], Jane Reznick[4], Martin Jastroch[1]

Affiliations

[1]Department of Molecular Biosciences, The Wenner-Gren Institute, Stockholm University, Stockholm, Sweden

[2]Department of Biology – Zoophysiology, Aarhus University, Denmark

[3]Biomedical Research Centre, Norwich Medical School, University of East Anglia, Norwich, UK

[4] University of Cologne, Faculty of Medicine and University Hospital Cologne, Cluster of Excellence Cellular Stress Responses in Aging-associated Diseases (CECAD), Cologne Germany

The PDF file includes:
Figs. S1 to S7

Supplemental figure captions

Figure S1. Chamber-based respirometry of isolated BAT mitochondria from the mouse and NMR in the presence of either pyruvate + malate or palmitoyl carnitine Data are presented as means ±SEM (n=3).

Figure S2. Blots with Ponceau S staining related to Figure 2A-B where mouse vs. NMR UCP1 content was determined from isolated BAT mitochondria.

Figure S3. Mouse and NMR wildtype UCP1 quantification from HEK293 stable cell line protein lysates using anti-UCP1 antibody (R&D Systems [MAB6158]). Standard means are plotted as black dots ±SD, whereas UCP1 levels from cell lysates are shown in green (mouse) or (blue). UCP1 levels were interpolated from a simple linear regression among the standards. The solid line indicates the regression line and the dotted lines represent the 95% confidence interval.

Figure S4. Plate-based respirometry traces of intact stable UCP1-expressing HEK cells where UCP1 variants are activated with arotinoid acid (TTNPB), or treated with a vehicle control (DMSO) which is denoted by red arrows. Black arrows denote the injections of oligomycin, DNP, and rotenone+antimycin A, respectively, from left to right. Traces have been corrected from non-mitochondrial respiration. Data are presented as means ±SEM (n=11-12 from 3 independent runs).

Figure S5. Permeabilized plate-based respirometry for all UCP1 variants stably overexpressed in HEK293 cells (related to Figure 4). Traces have been corrected from non-mitochondrial respiration. Data are presented as means ±SEM (n=10-12 from 3 independent runs).

Figure S6. Quantification of transiently expressed wildtype mouse vs. wildtype NMR UCP1 in HEK293 cells with purified standards using anti-UCP1 antibody (R&D Systems [MAB6158]).

Figure. S7. Plate-based respirometry traces of transiently transfected HEK293 cells overexpressing mouse UCP1 and NMR UCP1 variants, as well as the empty vector control. Black arrows denote the injections of oligomycin, DNP, and rotenone+antimycin A from left to right. Red arrow denotes the injection of palmitate or BSA vehicle control. Traces have been corrected from non-mitochondrial respiration. Data are presented as means ±SEM (n=7-9 from 4 independent runs).

Supplemental figures

Figure S1

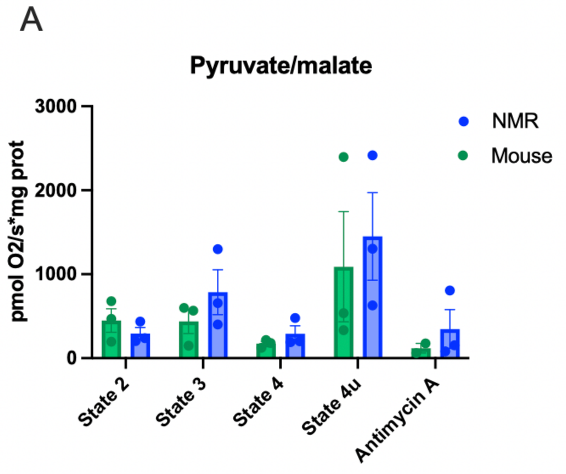

Figure S2.

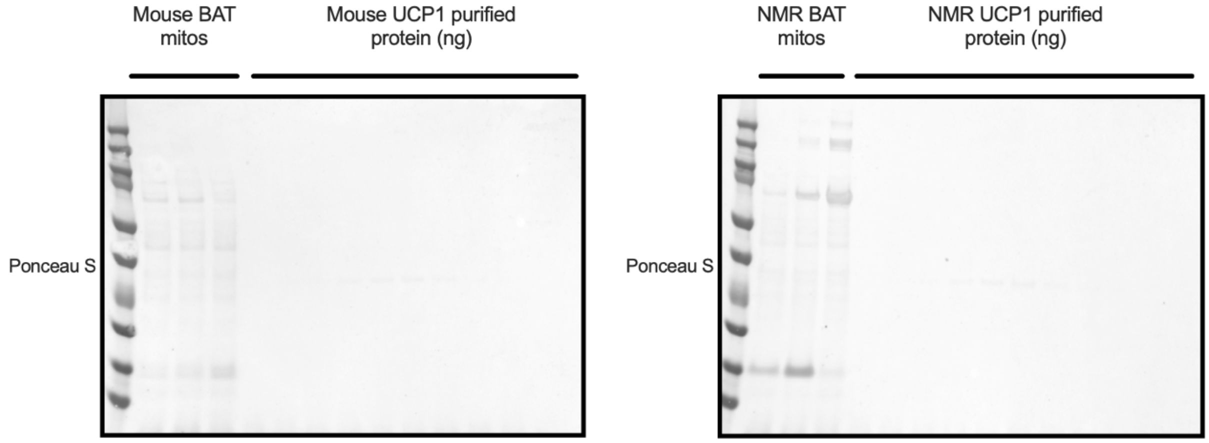

Figure S3.

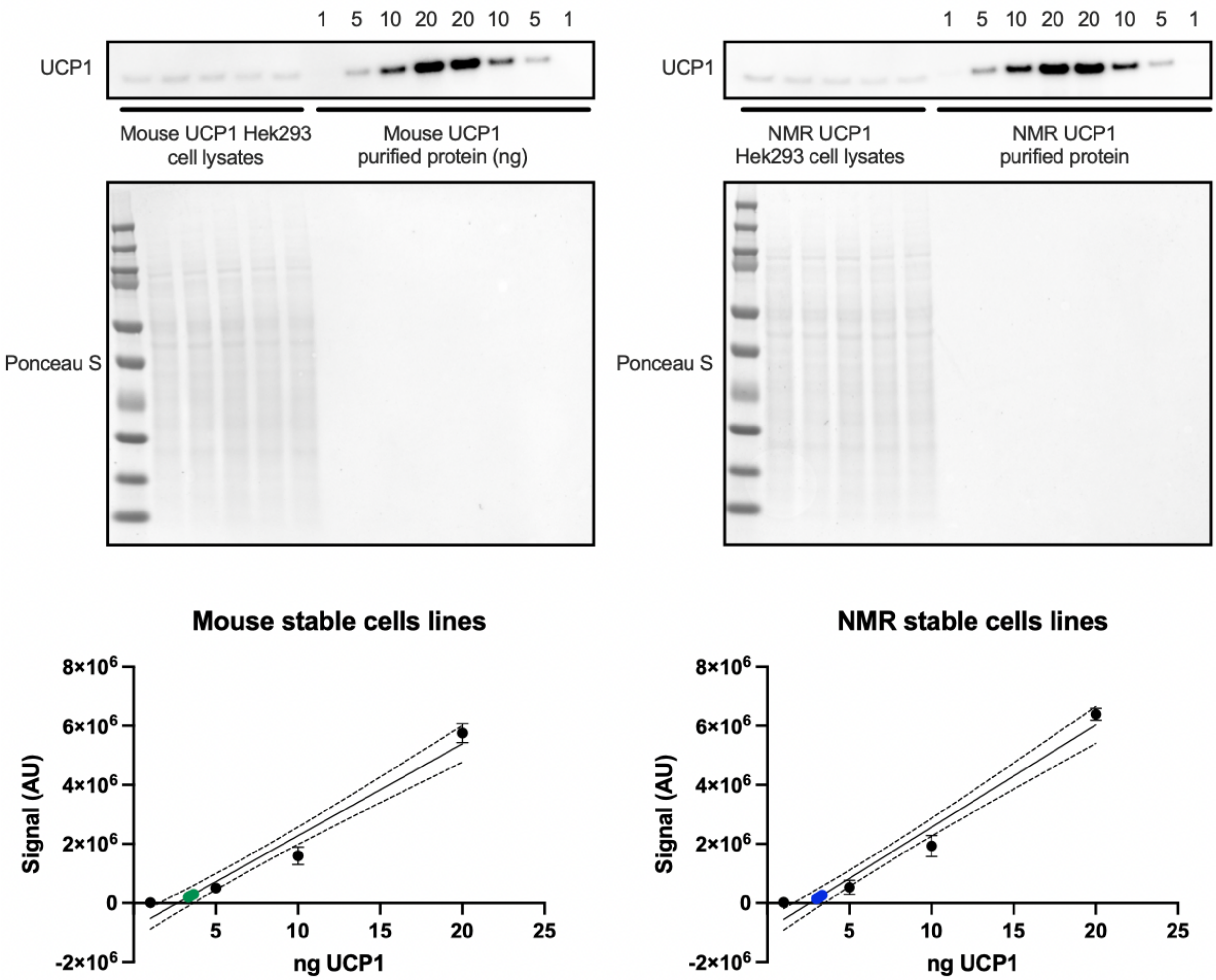
1 5 10 20 20 10 5 1
UCP1
Mouse UCP1 Hek293 cell lysates
Mouse UCP1 purified protein (ng)
Ponceau S
NMR UCP1 Hek293 cell lysates
NMR UCP1 purified protein
Mouse stable cells lines
NMR stable cells lines
Signal (AU)
8×10^6
6×10^6
4×10^6
2×10^6
0
-2×10^6
ng UCP1
5 10 15 20 25

Figure S4.

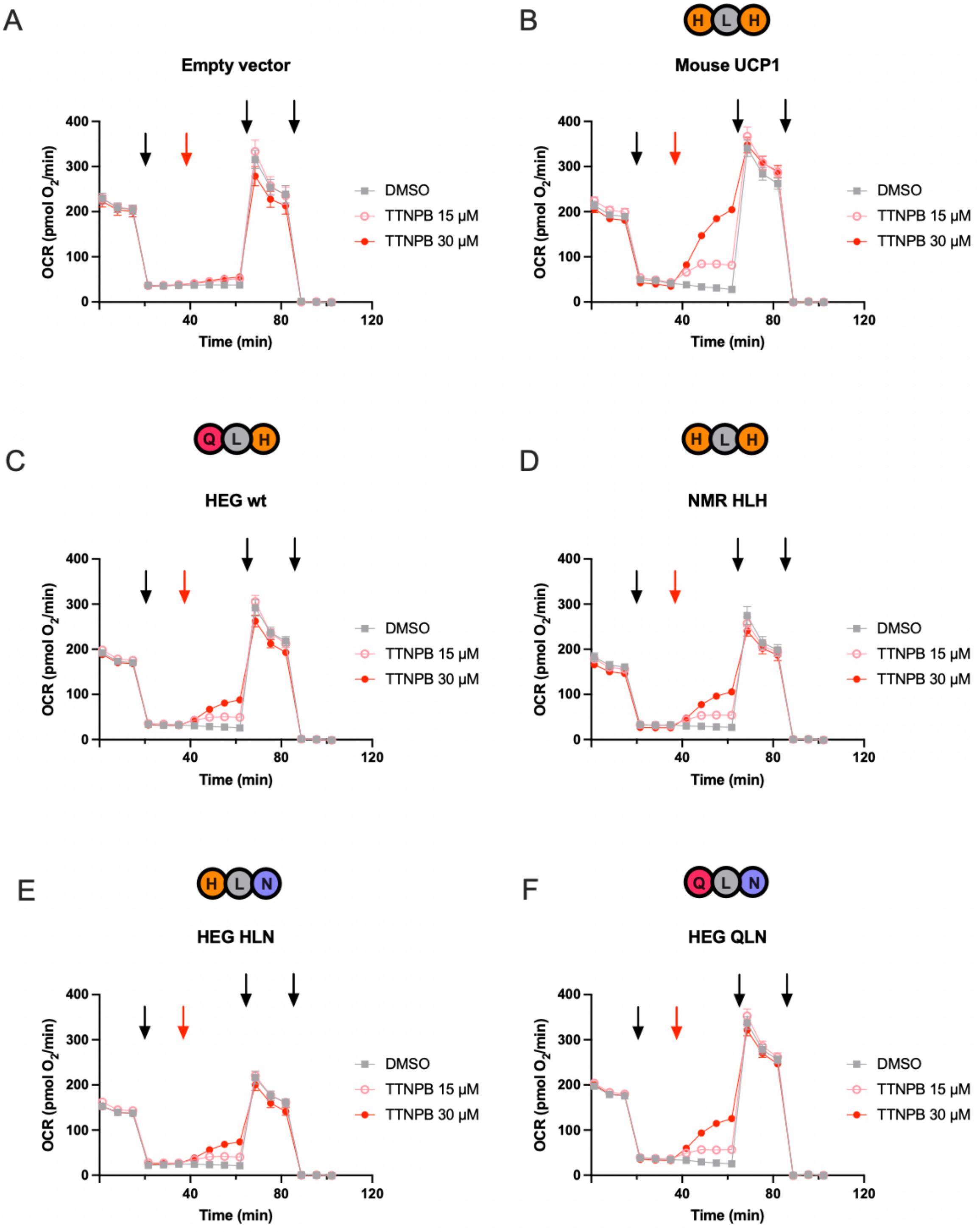
A
Empty vector
B
H L H
Mouse UCP1
C
Q L H
HEG wt
D
H L H
NMR HLH
E
H L N
HEG HLN
F
Q L N
HEG QLN
OCR (pmol O_2/min)
Time (min)
0
100
200
300
400
40
80
120
DMSO
TTNPB 15 μM
TTNPB 30 μM

Figure S5.

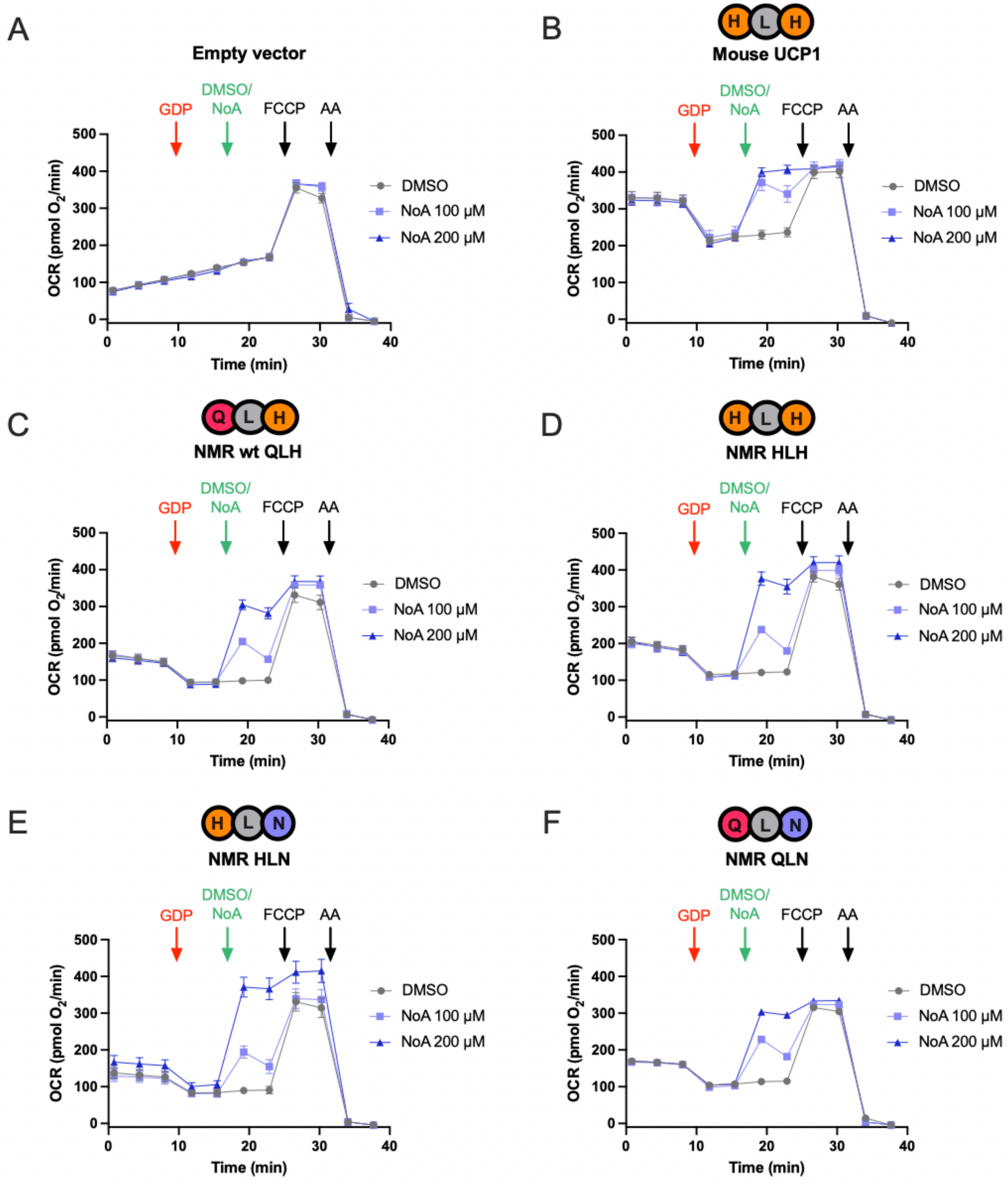

A
Empty vector
B
Mouse UCP1
C
NMR wt QLH
D
NMR HLH
E
NMR HLN
F
NMR QLN
GDP
DMSO/ NoA
FCCP
AA
OCR (pmol O2/min)
Time (min)
DMSO
NoA 100 μM
NoA 200 μM

Figure S6.

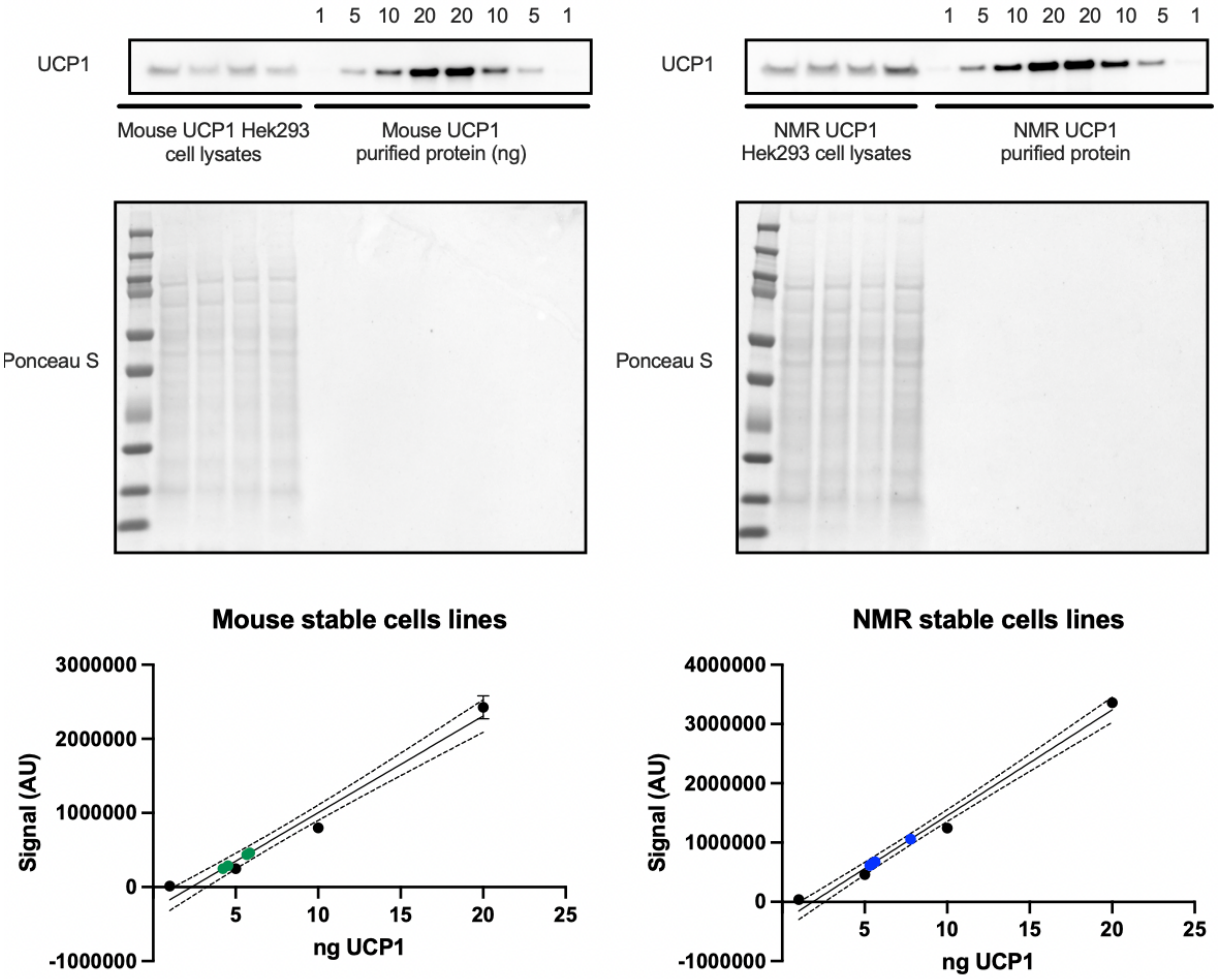

1 5 10 20 20 10 5 1
UCP1
Mouse UCP1 Hek293 cell lysates
Mouse UCP1 purified protein (ng)
Ponceau S
1 5 10 20 20 10 5 1
UCP1
NMR UCP1 Hek293 cell lysates
NMR UCP1 purified protein
Ponceau S
Mouse stable cells lines
Signal (AU)
3000000
2000000
1000000
0
-1000000
5
10
15
20
25
ng UCP1
NMR stable cells lines
Signal (AU)
4000000
3000000
2000000
1000000
0
-1000000
5
10
15
20
25
ng UCP1

Figure S7.

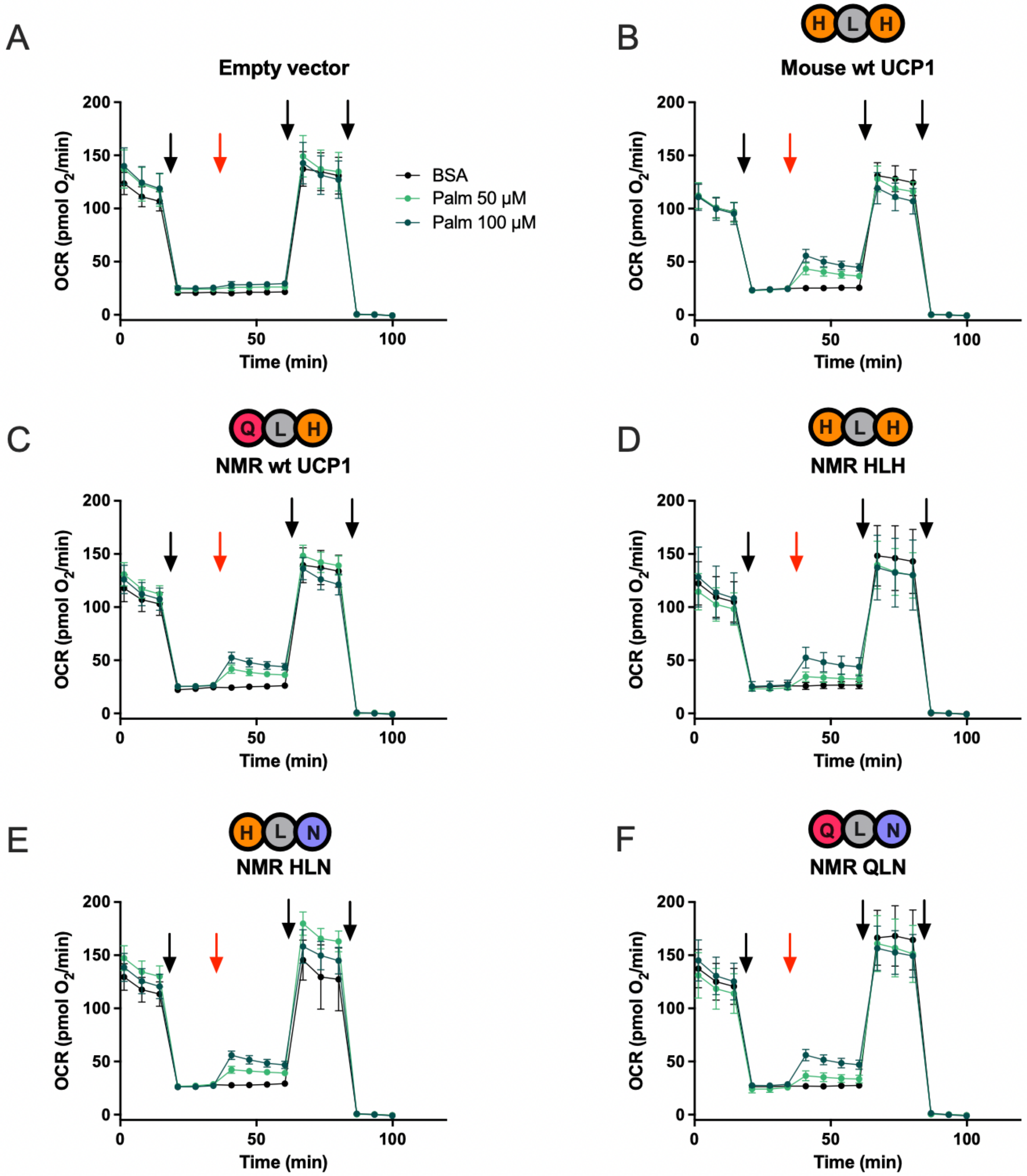
A
Empty vector
B
H L H
Mouse wt UCP1
C
Q L H
NMR wt UCP1
D
H L H
NMR HLH
E
H L N
NMR HLN
F
Q L N
NMR QLN
BSA
Palm 50 μM
Palm 100 μM
OCR (pmol O2/min)
Time (min)
0
50
100
150
200
